# Monosynaptic facilitation of motoneurons innervating intrinsic hand muscles mediated by group Ia afferents from the extensor carpi radialis in humans

**DOI:** 10.14814/phy2.15431

**Published:** 2022-08-26

**Authors:** Mitsuhiro Nito, Takuya Yoshimoto, Wataru Hashizume, Masaomi Shindo, Akira Naito

**Affiliations:** ^1^ Department of Anatomy and Structural Science Yamagata University School of Medicine Yamagata Japan; ^2^ Marunouchi Hospital Matsumoto Japan

**Keywords:** finger, group Ia afferents, monosynaptic facilitation, motoneuron, reflex, spinal cord, wrist

## Abstract

The projection pattern of low‐threshold afferents from the extensor carpi radialis (ECR) to motoneurons supplying intrinsic hand muscles was investigated using the post‐stimulus time‐histogram (PSTH) and electromyogram‐averaging (EMG‐A) methods. Electrical conditioning stimulation was applied to the radial nerve branch innervating the ECR. In the PSTH study, changes in the firing probability of single motor units following the stimulation were examined. An early and significant peak (facilitation) was induced in the motoneurons innervating the muscles, but the facilitation was induced infrequently. The central latency of the facilitation was equivalent to that of homonymous facilitation through monosynaptic path in the spinal cord. In the EMG‐A study, changes in the rectified and averaged electromyograms following the conditioning stimulation were examined. An early and significant peak (facilitation) was also induced. The facilitation disappeared after withdrawal of the vibration to the ECR muscle belly. Cutaneous nerve stimulation overlaying ECR never induced such facilitation in the PSTH and EMG‐A studies. These findings suggest that monosynaptic facilitation mediated by group Ia afferents of ECR to the motoneurons supplying intrinsic hand muscles exists in humans, but the connection seems to be weak. This weakness might allow manipulatory movements of the hand.

## Graphical abstract

The projection pattern of low‐threshold afferents from the extensor carpi radialis (ECR) to motoneurons supplying intrinsic hand muscles was investigated in healthy humans. Group Ia afferents of ECR increased the excitability of these motoneurons via monosynaptic path, but the connection seems to be weak. This weakness might allow manipulatory movements of the hand.

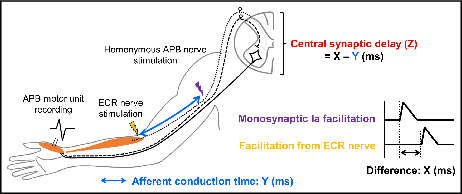

## 1 INTRODUCTION

Most daily activities involve hand grasping and manipulative movements. In these movements, the wrist is extended (Johanson et al., 1990; Nito et al., 2016), which is known as the tenodesis grasp (Kapandji, 1982). In addition, the wrist position is known to affect the grip strength (Lamoreaux & Hoffer, 1995; Li, 2002; O'Driscoll et al., 1992; Pryce, 1980), the maximal strength being exerted with a slight (about 20°) wrist extension. The wrist position also influences the pinch strength (Halpern & Fernandez, 1996; Lamoreaux & Hoffer, 1995). A spinal reflex arc mediated by group Ia afferents is one of the mechanisms supporting the movements. An excitatory reflex arc (facilitation) through a monosynaptic path assists the movements of agonistic muscles by increasing the excitability of the motoneurons. In contrast, an inhibitory reflex arc (inhibition) through an oligosynaptic (di or trisynaptic) path is known as reciprocal inhibition, and leads to the relaxation of antagonistic muscles by suppressing their excitability (Naito, 2003, 2004a, 2004b; Naito et al., 2012; Pierrot‐Deseilligny & Burke, 2012; Rothwell, 1994).

Previous studies using electrical conditioning stimulation of the median and ulnar nerves at the wrist showed that the extensor carpi radialis (ECR) motoneurons, which is a prime mover involved in wrist extension, received facilitation by group Ia afferents from intrinsic hand muscles through a monosynaptic path (Marchand‐Pauvert et al., 2000; Ogawa et al., 2005; Suzuki et al., 2005, 2012). The facilitation was also induced by weak mechanical tap stimulation to the thenar, first dorsal interosseous (FDI), and hypothenar muscles. Therefore, the reflex arcs may support the maintenance of the wrist extended position by increasing the excitability of ECR motoneurons. On the other hand, because wrist extension movements result in an increased grasping and pinch strength (Halpern & Fernandez, 1996; Lamoreaux & Hoffer, 1995; Li, 2002; O'Driscoll et al., 1992; Pryce, 1980), we hypothesized that the motoneurons supplying the intrinsic hand muscles receive facilitation from ECR afferents. The aim of this study was to explore the effects of the ECR on the excitability of motoneurons innervating the thenar (abductor pollicis brevis: APB), FDI, and hypothenar muscles (abductor digiti minimi: ADM).

## 2 MATERIALS AND METHODS

### 2.1 Subjects

A total of 14 healthy human volunteers (4 females and 10 males, 20–35 years old, right‐handed) participated in this study. None of the subjects had a history of neurological disease or were taking medication affecting the central nervous system. Experimental procedures were approved by the Ethics Committee of Yamagata University School of Medicine (approval number: 2019‐387) and were performed in accordance with the Declaration of Helsinki. Prior to participating in this study, all participants signed written informed consent to the experimental procedures.

### 2.2 Experimental setup

Two experiments were conducted in order to investigate (1) the effects on a single motor unit using the post‐stimulus time‐histogram (PSTH) method and (2) the effects on the motoneuron pool using the electromyogram‐averaging (EMG‐A) method. PSTH and EMG‐A studies were conducted on separate days. During the experiments, the subject sat comfortably in an armchair. The examined (right) arm lay on an armrest with the shoulder slightly flexed (about 20°) and the elbow semiflexed (about 45°).

### 2.3 Conditioning stimulation

Rectangular electrical pulses of 1.0 ms duration were delivered percutaneously to the radial nerve branch innervating the ECR (ECR nerve) using bipolar surface electrodes (0.8 cm diameter, 1.5 cm interelectrode distance). The stimulus electrodes were placed on the skin overlaying the motor point of ECR muscle, which was defined as the location where muscle contraction could be evoked with the minimum stimulus intensity, and were connected to an electrical stimulator (SEN‐7203, Nihon Kohden) through an isolator (SS‐104 J, Nihon Kohden). A stimulus intensity just below the threshold of direct motor response (M‐wave) of ECR was used. The M‐wave was recorded with a pair of Ag/AgCl disk surface electrodes (0.8 cm diameter, NT‐211U; Nihon Kohden), and the stimulus intensity was expressed as multiples of the threshold of the M‐wave [1.0 × motor threshold (MT)]. This stimulus intensity was used to investigate the effect of group I afferents, as they have a lower threshold than alpha motor axons (Kandel et al., 2012; Rothwell, 1994). To confirm that we stimulated the nerve branch innervating the ECR and not the muscle tissue itself, the stimulating position was carefully selected so that a small stepwise increase of the stimulus intensity from 1.0 × MT resulted in a rapid increase in the amplitude of the M‐response of ECR. The absence of other muscle contraction in response to intensities above 2.5 × MT was checked by palpation. The recording electrodes were also used to confirm that the ECR muscle was in resting condition. To investigate the effect of cutaneous afferents, an electrical stimulation was applied to the skin using the bipolar surface electrodes placed 0.5–1.0 cm laterally from the ECR nerve. The stimulus intensity for cutaneous afferents was adjusted to the same voltage than the ECR nerve stimulation.

### 2.4 PSTH study

We investigated the effect of conditioning stimulation on a single motor unit using the PSTH method (Fournier et al., 1986). Motor unit discharges were recorded using a pair of needle electrodes (Seirin acupuncture needle, 0.16 mm diameter, Seirin Kasei) inserted into the muscle belly of APB, FDI, and ADM. APB and ADM are part of the thenar and hypothenar muscles, respectively. EMG signals were amplified (×5000), and processed with a low‐cut filter (50 Hz).

PSTHs (bin width 0.2 or 0.5 ms) of the discharge of a voluntarily activated motor unit (firing interval: about 100 ms) were constructed for the period ranging from 10 to 50 ms after the conditioning stimulation. The subject was requested to perform static (isometric) contractions of the recorded muscle. The APB motor unit discharges were recorded during thumb abduction with the forearm supinated, those of FDI were obtained by index finger abduction with the forearm pronated, and little finger abduction with the forearm supinated allowed to record ADM motor unit discharges. Auditory and visual feedbacks of the EMG potentials were provided to help the subjects to maintain stable activities of single motor units. A single motor unit potential was amplified, and it was displayed on an oscilloscope (TDS210, Tektronix) and connected to a loudspeaker as well. The subject could thus recognize the constant shape of the motor unit, and at the same time could hear the sound of regular firing of the motor unit during weak voluntary contraction. Discrimination of a single motor unit during tonic voluntary contractions was carefully determined by an upper or lower discriminating level of the potential. The EMG potentials were converted into standard pulses, which were used to trigger a computer that subsequently activated the stimulator about every 0.7 s. The stimulation was triggered with a delay of about 70 ms after the voluntary activation of the motor unit. The delay was set at a time that easily affected the next motor unit firing. In other words, the delay was set so that the afferent volleys by electrical stimulation would arrive at the motoneuron around the latest period of hyperpolarization due to the previous motor unit firing and just before voluntarily‐driven discharge of the motoneuron. The delay was determined by taking into consideration the firing interval of the voluntarily activated motor unit. A histogram of firing probability was also constructed in a control situation without stimulation. The control and stimulated situations were alternated randomly (same number of triggers) within a sequence. Each situation was generally compiled from the responses to approximately 200–1000 triggers, although the number of triggers was increased in some cases above 1000 triggers to clarify the facilitatory effect. To show the effects of the conditioning stimulation, the number of triggers in each bin in the control situation was subtracted from that obtained after stimulation. Cumulative sum curve was constructed by the subtracted histograms in order to confirm the facilitatory effect (Ellaway, 1978).

**FIGURE 1:**
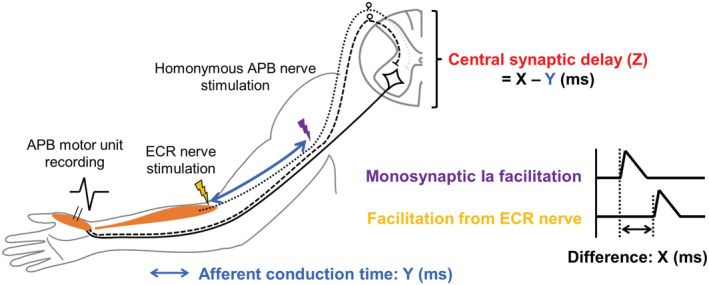
Schematic drawings of an experimental design with a post‐stimulus time‐histogram study for estimation of the central latency. This schema shows an example of the effect induced by stimulation to the radial nerve branch innervating the extensor carpi radialis (ECR nerve) and that by stimulation to the homonymous afferents of the abductor pollicis brevis (APB) at the axilla on a single APB motor unit.

Next, we analyzed the facilitation of motor units induced by ECR afferents. To estimate the central synaptic delay of the process, the latencies of the facilitation and the monosynaptic peak in PSTH following stimuli of the respective ECR and homonymous afferents from intrinsic hand muscles (APB, FDI, ADM) were compared (Cavallari et al., 1992; Cavallari & Katz, 1989; Katz et al., 1991; Kobayashi et al., 2016; Marchand‐Pauvert et al., 2000; Miyasaka et al., 2007; Naito et al., 1996, 1998; Nakano et al., 2014; Nito et al., 2016; Nito, Hashizume, Jimenji, et al., 2018; Sato et al., 2018; Shinozaki et al., 2017) (Figure 1). To investigate the effect on APB motor units, APB homonymous afferents were stimulated at the proximal part of the medial intermuscular septum of the arm (axilla) with bipolar surface electrodes (0.8 cm diameter, 1.5 cm interelectrode distance). Because the efferent conduction times for the facilitation of APB by ECR afferents and for the homonymous facilitation of APB were identical, the difference between the two latencies (*X*) reflected the afferent conduction time between the two stimulation sites (*Y*) and the central synaptic delay (*Z*) (*X* = *Y* + *Z*). The afferent conduction time (*Y*) was measured using an ECR motor unit as the difference between two latencies of homonymous monosynaptic Ia facilitation induced by stimulation of the ECR nerve and the axilla. The central synaptic delay (Z) of the effect was obtained by subtracting *Y* from *X* (*Z* = X − Y). The central synaptic delay was estimated using a similar technique for the investigation of the effects on FDI and ADM motor units.

### 2.5 EMG‐A study

We investigated the effect of conditioning stimulation on the motoneuron pool using the EMG‐A method (Capaday et al., 1990; Meunier et al., 1996; Nito et al., 2016; Nito, Hashizume, Jimenji, et al., 2018; Nito, Hashizume, Suzuki, et al., 2018; Ogawa et al., 2005; Petersen et al., 1998, 1999; Sato et al., 2018; Suzuki et al., 2005, 2012). The EMG signals were recorded with the pair of Ag/AgCl disk surface electrodes mentioned above. The electrodes were secured to the skin over each muscle belly. A ground electrode (wet gauze) put around the forearm was used as the reference. EMG signals were amplified (×2000), bandpass filtered (15–1000 Hz), and sampled at 0.2 ms.

To obtain EMG activities, the subject performed weak and static contractions at 5% of the maximal EMG activity recorded during maximal voluntary contraction as in the PSTH study. At the beginning of the experiment, the contraction level was determined by the ratio (percentage) to the amplitude of rectified and integrated EMGs recorded during maximum voluntary contraction and that was displayed on an oscilloscope (VC‐6723; Hitachi). The EMG signals were rectified and averaged by a personal computer using an EMG‐averaging program (TeraAve MTS00140, Gigatex). To prevent muscle fatigue, the experiment was divided into several sequences comprising of 50 to 100 stimuli. The EMGs obtained in these sequences were added and averaged. The conditioning stimulation was delivered at random intervals of 0.8 to 1.2 s during recording of EMG activities. Changes in the waveform of the averaged EMG following the stimulation were investigated.

To examine the influence of tonic vibration stimulation (TVS), which is known to affect the transmission of group Ia afferents (Coppin et al., 1970; Fetz et al., 1979; Hayward et al., 1986; Nito et al., 2021), on the facilitation induced by the conditioning stimulation, TVS at 100 Hz was delivered to the ECR muscle belly for 6 min using a portable vibrator (MD‐011, Daito Electric Machine) (Nito et al., 2021). TVS is known to decrease postsynaptic potentials evoked by Ia afferents in spinal motoneurons and the depression was probably due to reduction in number of Ia afferents participating in the facilitation (Coppin et al., 1970; Fetz et al., 1979; Hayward et al., 1986; Nito et al., 2021). The head of the vibrator was firmly covered by a thermoplastic material (Ezeform Splinting Material, Sakai Medical), on which a steel screw bar (0.5 cm diameter, 5.0 cm length) was installed. A small bell‐shaped plastic head (1.0 cm diameter, 2.0 cm length) was fitted to the tip of the screw bar. The plastic head was carefully pressed to the ECR muscle belly, the experimenter's hand holding the vibrator. During TVS, the subject stayed in resting condition. The intensity of TVS was adjusted by changing pressure of the head against the muscle for each subject and was set to be just subthreshold for a tonic vibration reflex.

Changes in the rectified and averaged EMG activities after the conditioning stimulation were compared between situations with and without TVS. The EMGs were recorded before (Pre) and after the removal of TVS, every 10 or 15 min. In order to clarify the time course of the TVS effect, data were obtained with fewer conditioning stimuli (300–600 stimuli) than those in the other experiments (about 1000 stimuli). The latency and duration of the effect were determined from the waveform obtained in experiments with many (about 1000) stimuli. This time window was also used as analysis time window for the TVS study.

### 2.6 Data and statistical analyses

In the PSTH study, the latency and duration of the effect were defined as consecutive bins constructed by an increase or decrease in firing probability and judged from a fluctuation of the cumulative sums by visual inspection. To determine whether the firing probabilities after stimulation differed from those obtained in the control situation for the time window consisting of several consecutive bins, *χ* ^2^‐test was used. These analyses were done using a personal computer with a PSTH analysis program (MTS0014, Gigatex).

In the EMG‐A study, the mean amplitude of the effect after the stimuli was compared with those measured 10 to 60 ms before the stimuli (100%, control) for each subject using Student's *t*‐test. To identify the period of the effect by the conditioning stimulation, the mean and standard deviation (SD) of the amplitude of rectified and averaged EMGs were calculated for the control period before the stimulation and rectified and averaged EMGs exceeding 3 SD from the control level were considered as an effect of the stimulation. Since the rectified and averaged EMG exceeded ±3SD lines in the middle of trajectory of the effect, the very onset and offset of the effect were determined by visual inspection (Nito, Hashizume, Suzuki, et al., 2018). The amount of facilitation was calculated as a difference between the effect and the control value (100%). To examine TVS effects, one‐way ANOVA with Games‐Howell post hoc tests was used. Statistical significance was considered for *p* < 0.05. Statistical analyses were performed using SPSS 26 (IBM).

## 3 RESULTS

### 3.1 PSTH study

#### 3.1.1 Effects on the APB motor units

**FIGURE 2:**
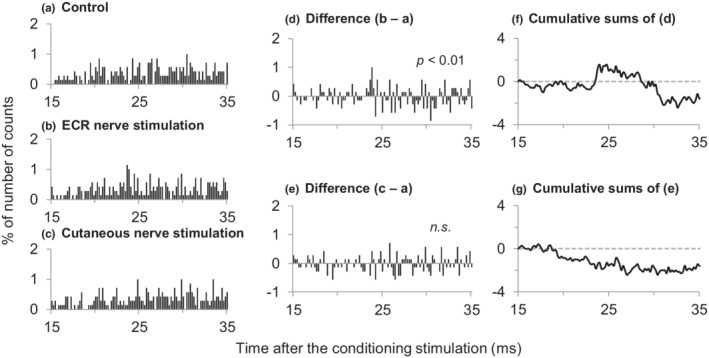
Changes in firing probability of an abductor pollicis brevis (APB) motor unit following stimulation of the radial nerve innervating the extensor carpi radialis (ECR nerve) and the cutaneous nerve. Time histograms obtained without stimulation (a), with stimulation of ECR (b) and cutaneous nerves (c) within a sequence. The number of triggers was 700 (a–c). Each column of (d) and (e) represents the difference between the situations with and without stimulation. Cumulative sums of (f) and (g) were obtained from each subtracted histogram of (d) and (e), respectively. Ordinates represent the number of counts as a percentage of the numbers of triggers. Abscissae represent the latency after stimulation (0.2 ms bins).

Figure 2a–d shows a representative example of facilitation. A significant peak was observed 23.2 to 24.0 ms after the ECR nerve stimulation with an intensity of 0.98 × MT (*p* < 0.01). The duration of the peak was 0.8 ms. Sixty‐four APB motor units were examined in five subjects with 3–15 measurements in each subject. An early and significant (*p* < 0.05 for 3 motor units; *p* < 0.01 for 11 motor units) peak (an increase in the firing probability, facilitation) was induced by the ECR nerve stimulation in 14 motor units (22%). The remaining 50 motor units showed neither significant peak nor trough. The latency and duration of the peaks ranged from 22.0 to 35.6 ms (mean ± SD: 26.2 ± 3.3 ms) and from 0.8 to 2.8 ms (1.6 ± 0.5 ms), respectively. Effect of cutaneous nerve stimulation was examined in 6 of 14 motor units obtained from 5 subjects, and the stimulation did not induce such peak (Figure 2).

**FIGURE 3:**
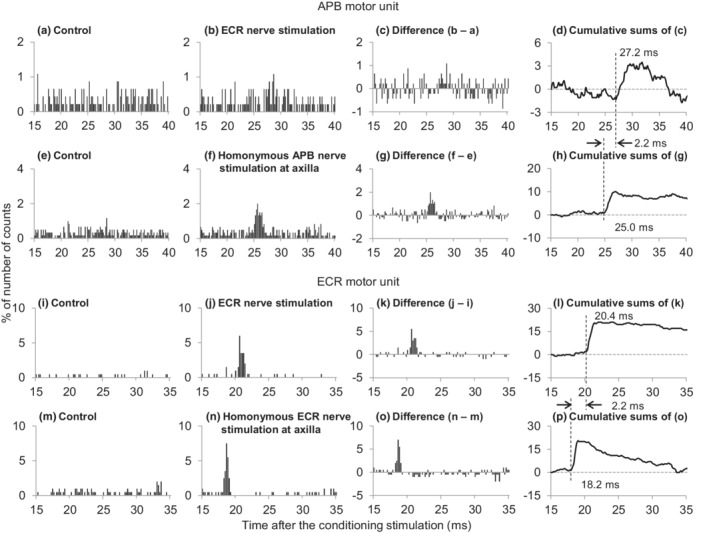
Changes in firing probability of the abductor pollicis brevis (APB, a–h) and extensor carpi radialis (ECR) motor units (i–p) following stimulation of the radial nerve innervating ECR and each homonymous nerve. Time histograms obtained in the control situation without stimulus (a, e, i, m) and after stimulation of the ECR (b, j) or the homonymous APB (f) or ECR nerves (n). Differences between the histograms obtained in control and stimulated conditions (c, g, k, o). The number of triggers were 460 (a, b), 600 (e, f), 200 (i, j), and 200 (m, n). Cumulative sums were obtained from the subtracted histograms. Ordinates represent the number of counts as a percentage of the numbers of triggers. Abscissae represent the latency after stimulation (0.2 ms bins). Vertical dashed lines indicate the onset latencies of the facilitation.

Figure 3 shows part of the process of estimating the central synaptic delay of the facilitation in a representative subject. The difference between the latencies of the facilitation induced by the ECR nerve stimulation (Figure 3a–d) and the facilitation induced by the homonymous APB afferents stimulation at the axilla (Figure 3e–h) in a single APB motor unit was 2.2 ms. Next, we examined the afferent conduction time of ECR afferents while recording a single ECR motor unit instead of APB. From the latency difference between the homonymous facilitation of ECR induced by stimulation of the ECR nerve and that by stimulation of the axilla, the afferent conduction time of the ECR nerve was 2.2 ms (Figure 3i–p). Hence, the central synaptic delay of the facilitation was estimated to be 0.0 ms (=2.2–2.2 ms) longer than that of the homonymous Ia facilitation. The process of the central synaptic delay was examined in 8 of 14 APB motor units obtained from the five subjects (Table 1) and ranged a −0.6 to 0.8 ms (0.1 ± 0.4 ms) longer delay compared with that of the monosynaptic Ia facilitation.

**TABLE 1:** The excess of the central synaptic delay of the facilitation from the extensor carpi radialis (ECR) to abductor pollicis brevis (APB) over that of the homonymous facilitation in APB motor units

Subject	Motor unit	Onset latency (ms)		Difference (ms)	Distance between two stimulation sites (m)	Conduction time (ms)	Excess of central synaptic delay (ms)
		Facilitation from ECR to APB	Homonymous facilitation of APB	(*X*)		(*Y*)	(*X*−*Y*)
A	A1	27.0	25.0	2.0	0.20	1.8	0.2
B	B1	23.4	20.8	2.6	0.20	2.6	0.0
	B2	26.4	23.0	3.4	0.20	3.0	0.4
C	C1	22.0	18.6	3.4	0.19	2.6	0.8
D	D1	24.8	23.0	1.8	0.20	2.4	−0.6
	D2	26.8	24.6	2.2	0.21	2.6	−0.4
E	E1	27.6	25.0	2.6	0.20	2.8	−0.2
	E2	27.0	24.6	2.4	0.20	2.2	0.2
						Mean ± SD	0.1 ± 0.4

#### 3.1.2 Effects on the FDI motor units

**FIGURE 4:**
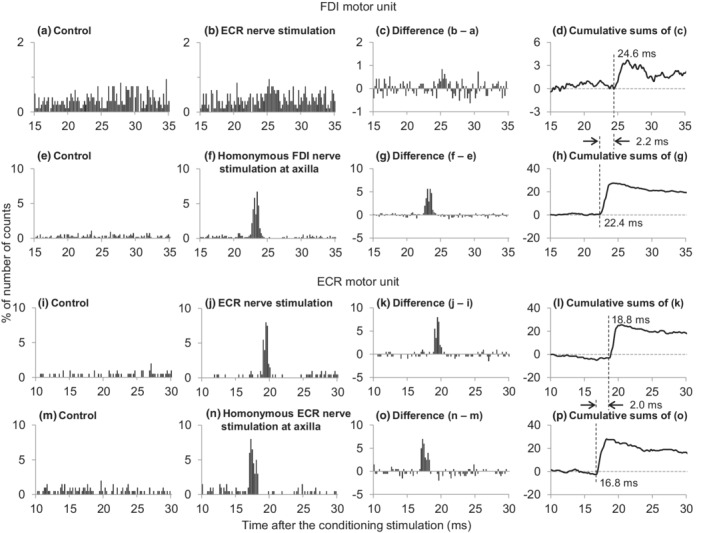
Changes in firing probability of the first dorsal interosseous (FDI, a–h) and extensor carpi radialis (ECR) motor units (i–p) following stimulation of the radial nerve innervating ECR and each homonymous nerve. Time histograms obtained in the control situation without stimuli (a, e, i, m) and after stimuli of the ECR (b, j) or the homonymous FDI (f) and ECR nerves (n). Differences between the histograms obtained in control and stimulated conditions (c, g, k, o). The number of triggers were 950 (a, b), 550 (e, f), 200 (i, j), and 200 (m, n). Cumulative sums were obtained from the subtracted histograms. Ordinates represent the number of counts as a percentage of the numbers of triggers. Abscissae represent the latency after stimulation (0.2 ms bins). Vertical dashed lines indicate the onset latencies of the facilitation.

Forty‐one FDI motor units were examined in five subjects with 6–18 measurements in each subject. An early and significant (*p* < 0.05 for 3 motor units; *p* < 0.01 for 9 motor units; *p* < 0.001 for 3 motor units) peak (facilitation) was induced by the ECR nerve stimulation in 15 motor units (37%) (Figure 4). No significant peak was observed for the remaining 26 motor units. The latency and duration of the peak ranged from 21.0 to 33.4 ms (25.8 ± 3.1 ms) and 0.6 to 6.2 ms (2.6 ± 1.6 ms), respectively. Effect of cutaneous nerve stimulation was examined in 5 of 15 motor units obtained from 5 subjects, and the stimulation did not induce such peak.

The process of the central synaptic delay was examined in 9 of 15 FDI motor units obtained from the five subjects (Table 2) and ranged a −0.6 to 0.5 ms (−0.1 ± 0.4 ms) longer delay compared with than that of the monosynaptic Ia facilitation.

**TABLE 2:** The excess of the central synaptic delay of the facilitation from the extensor carpi radialis (ECR) to first dorsal interosseous (FDI) over that of the homonymous facilitation in FDI motor units

Subject	Motor unit	Onset latency (ms)		Difference (ms)	Distance between two stimulation sites (m)	Conduction time (ms)	Excess of central synaptic delay (ms)
		Facilitation from ECR to FDI	Homonymous facilitation of FDI	(*X*)		(*Y*)	(*X* − *Y*)
A	A1	26.8	23.5	3.3	0.19	2.8	0.5
	A2	27.6	25.0	2.6	0.24	3.2	−0.6
B	B1	22.8	20.2	2.6	0.2	3.0	−0.4
	B2	24.6	22.4	2.2	0.22	2.4	−0.2
	B3	27.2	23.4	3.8	0.20	3.8	0.0
	B4	27.6	25.2	2.4	0.22	2.0	0.4
C	C1	28.0	26.0	2.0	0.24	2.2	−0.2
F	F1	24.4	22.0	2.4	0.18	2.6	−0.2
G	G1	25.0	21.8	3.2	0.22	3.2	0.0
						Mean ± SD	−0.1 ± 0.4

#### 3.1.3 Effects on the ADM motor units

**FIGURE 5:**
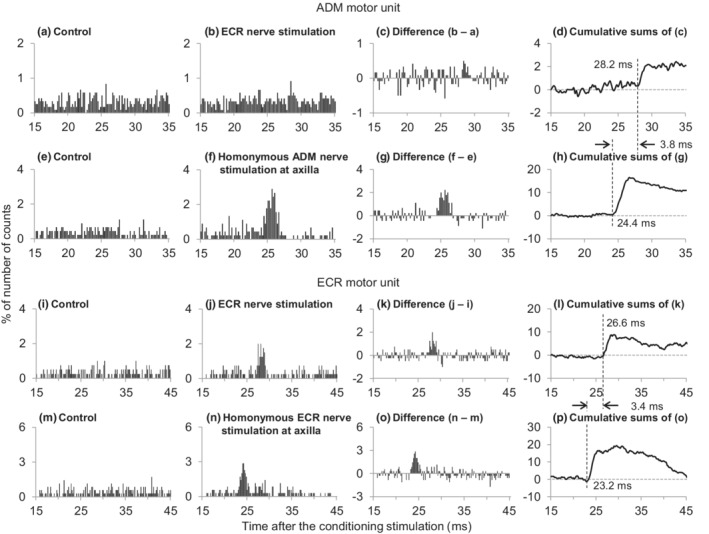
Changes in firing probability of the abductor digiti minimi (ADM, a–h) and extensor carpi radialis (ECR) motor units (i–p) following stimulation of the radial nerve innervating ECR and each homonymous nerve. Time histograms obtained in the control situation without stimuli (a, e, i, m) and after stimuli of the ECR (b, j) or the homonymous ADM (f) or ECR nerves (n). Differences between the histograms obtained in control and stimulated conditions (c, g, k, o). The numbers of triggers were 1200 (a, b), 450 (e, f), 400 (i, j), and 350 (m, n). Cumulative sums were obtained from the subtracted histograms. Ordinates represent the number of counts as a percentage of the numbers of triggers. Abscissae represent the latency after stimulation (0.2 ms bins). Vertical dashed lines indicate the onset latencies of the facilitation.

Thirty‐eight ADM motor units were examined in six subjects with 4–11 measurements in each subject. An early and significant (*p* < 0.05 for 3 motor units; *p* < 0.01 for 9 motor units; *p* < 0.001 for 2 motor units) peak was induced by the ECR nerve stimulation in 14 motor units (37%) (Figure 5), whereas no significant peak was obtained for the remaining 24 motor units. The latency and duration of the peak ranged from 24.0 to 31.4 ms (27.3 ± 2.2 ms) and 0.6 to 4.0 ms (1.9 ± 1.0 ms), respectively. Effect of cutaneous nerve stimulation was examined in 5 of 14 motor units obtained from 5 subjects, and the stimulation did not induce such peak.

The process of the central synaptic delay was examined in 10 of 14 ADM motor units obtained from the five subjects (Table 3) and ranged a − 0.6 to 0.5 ms (0.0 ± 0.3 ms) longer delay compared with than that of the monosynaptic Ia facilitation.

**TABLE 3:** The excess of the central synaptic delay of the facilitation from the extensor carpi radialis (ECR) to abductor digiti minimi (ADM) over that of the homonymous facilitation in ADM motor units

Subject	Motor unit	Onset latency (ms)		Difference (ms)	Distance between two stimulation sites (m)	Conduction time (ms)	Excess of central synaptic delay (ms)
		Facilitation from ECR to ADM	Homonymous facilitation of ADM	(*X*)		(*Y*)	(*X* − *Y*)
A	A1	25.4	23.6	1.8	0.24	2.4	−0.6
	A2	27.6	24.4	3.2	0.22	2.8	0.5
	A3	28.2	24.4	3.8	0.24	3.4	0.4
B	B1	24.0	20.0	4.0	0.24	4.2	−0.2
	B2	24.0	20.2	3.8	0.25	4.0	−0.2
	B3	29.0	25.2	3.8	0.22	3.8	0.0
C	C1	26.4	22.8	3.6	0.26	3.6	0.0
F	F1	27.4	25.0	2.4	0.18	2.2	0.2
G	G1	27.0	24.0	3.0	0.23	3.1	−0.1
	G2	31.4	27.4	3.0	0.21	2.6	0.4
						Mean ± SD	0.0 ± 0.3

### 3.2 EMG‐A study

#### 3.2.1 Effects on the APB motoneuron pool

**FIGURE 6:**
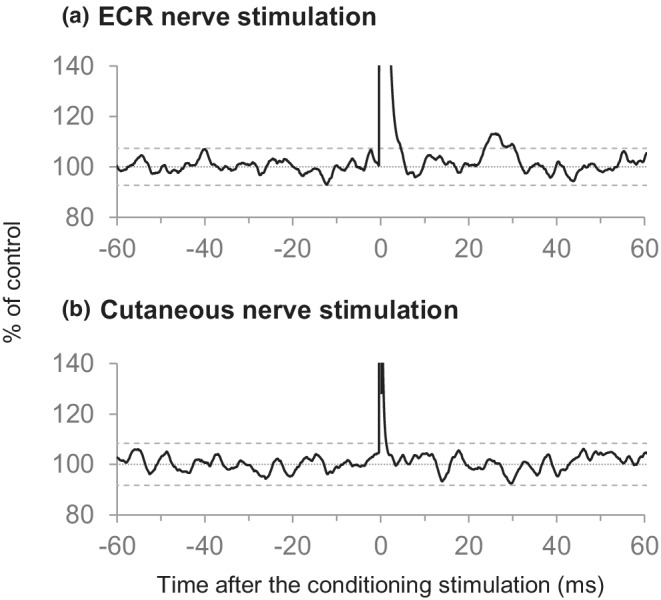
Changes in the rectified and averaged electromyograms of the abductor pollicis brevis (APB) following stimulation of the radial nerve innervating the extensor carpi radialis (ECR nerve) and cutaneous nerve. Representative waveforms were obtained from a single subject. ECR nerve stimulation induced an early and significant peak in rectified and averaged electromyograms of APB (a). In contrast, cutaneous nerve stimulation did not induce any significantly effect (b). Each waveform was obtained by 800 sweeps. Horizontal dashed lines indicate the ±3 SD calculated from the control period before the conditioning stimulation.

The ECR nerve stimulation induced an early and significant peak (facilitation) in rectified and averaged EMGs of APB in all 10 subjects (*p* < 0.05 for five subjects; *p* < 0.01 for four subjects; *p* < 0.001 for one subject) (Table 4, Figure 6). The amplitude of the peak was 112.3% ± 1.4% (peak value), the amount of facilitation being 12.3% ± 1.4%. The latency and duration of the peak were 24.2 ± 1.6 ms and 12.6 ± 2.3 ms, respectively. For each subject, the peak observed in the PSTH study was within the time range in which the peak was recorded in the EMG‐A study. Such peak was never induced by electrical stimulation of the skin overlaying the ECR nerve in all the subjects (Figure 6).

**TABLE 4:** Facilitation from extensor carpi radialis to motoneuron pools supplying the abductor policis brevis (APB), first dorsal interosseous (FDI), and abductor digiti minimi (ADM)

Motoneurons	Subject	Latency (ms)	Duration (ms)	Amount of facilitation (%)		Statistics
				Peak value	Mean value	
APB	A	24.6	16.8	12.1	7.8	**
	B	23.2	10.8	12.1	6.0	**
	C	21.8	11.0	13.2	7.2	**
	D	23.4	13.2	10.3	5.4	*
	E	26.4	13.4	14.5	7.0	***
	G	22.2	12.8	13.7	7.0	**
	H	26.0	14.8	10.4	5.8	*
	I	25.0	13.6	11.4	7.3	*
	J	25.4	8.8	13.6	7.2	*
	K	24.4	10.4	11.6	6.5	*
	Mean ± SD	24.2 ± 1.6	12.6 ± 2.3	12.3 ± 1.4	6.7 ± 0.8	
FDI	A	22.0	8.0	17.5	8.5	**
	B	21.2	15.0	15.5	7.6	***
	C	20.6	9.4	11.7	7.6	**
	F	24.4	11.2	15.3	7.8	**
	G	24.0	8.8	20.9	10.2	**
	H	22.4	9.2	18.2	7.1	**
	I	26.4	11.2	15.9	6.7	*
	L	21.6	16.8	9.7	5.1	**
	M	23.4	7.4	10.3	6.3	**
	N	27.6	12.4	11.0	7.0	**
	Mean ± SD	23.4 ± 2.3	10.9 ± 3.1	14.6 ± 3.8	7.4 ± 1.4	
ADM	A	24.6	7.8	15.2	7.9	**
	B	22.0	11.2	13.7	7.7	**
	C	24.0	12.0	12.1	5.5	*
	E	24.4	13.4	9.6	4.4	*
	F	24.0	15.4	13.0	7.1	**
	G	24.4	6.4	11.2	6.1	*
	H	23.8	16.0	7.5	4.9	*
	I	26.4	8.8	11.0	4.8	*
	K	26.0	10.4	10.5	6.0	*
	N	26.2	14.8	11.6	6.3	*
	Mean ± SD	24.6 ± 1.3	11.6 ± 3.3	11.5 ± 2.2	6.1 ± 1.2	

* *p* < 0.05.

** *p* < 0.01.

*** *p* < 0.001.

**FIGURE 7:**
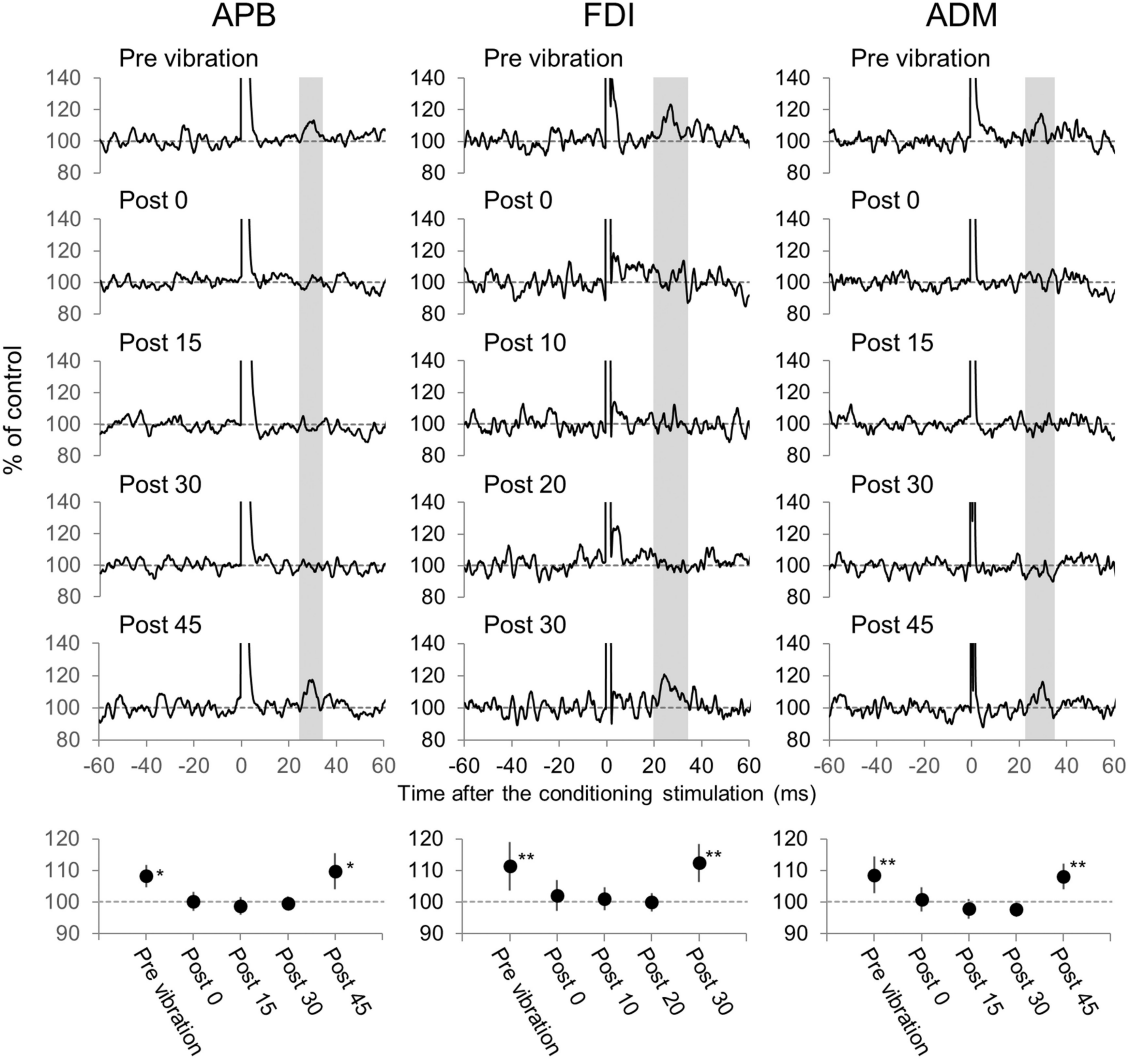
Changes in the rectified and averaged electromyograms from various hand muscles following stimulation of the radial nerve innervating the extensor carpi radialis. The upper panel shows that representative waveforms were obtained from individual subjects by rectified and averaged electromyograms of 600 sweeps in the abductor pollicis brevis (APB), 350 in the first dorsal interosseous (FDI), and 500 in the abductor digiti minimi (ADM). The analysis time window (gray area) was determined from the waveform of the facilitation, which was obtained after many stimuli (not shown). The beginning and end of this area indicates the onset and offset of the facilitation, respectively. The lower panel shows the changes in the peak mean amplitude before and after the vibration during the analysis time window. The asterisks indicate a significant difference of peak mean amplitude compared with that measured during the control period (before the conditioning stimulation). *p < 0.05, **p < 0.01.

Figure 7 shows the influence of TVS on the peak induced by ECR nerve stimulation. The peak (mean value: 8.2%, *p* < 0.05) disappeared almost completely after withdrawal of TVS, and was recovered 45 min after TVS (9.7%, *p* < 0.05). These results were supported by the ANOVA indicating a significant effect (*F*_4,97.778_ = 76.947, *p* < 0.001). Post hoc tests revealed that no change was observed at 45 min after removal of TVS when compared to the mean value of facilitation before TVS (*p* = 0.621). The influence of TVS was examined in five out of ten subjects. The peak diminished following TVS and was recovered 30–45 min (38 ± 8 min) after TVS cessation in all the subjects.

#### 3.2.2 Effects on the FDI motoneuron pool

The ECR nerve stimulation induced an early and significant peak (facilitation) in rectified and averaged EMGs of FDI in all 10 subjects (*p* < 0.05 for one subject; *p* < 0.01 for eight subjects; *p* < 0.001 for one subject) (Table 4, Figure 7). The amount of facilitation was 14.6% ± 3.8%. The latency and duration of the peak were 23.4 ± 2.3 ms and 10.9 ± 3.1 ms, respectively. The peak in the PSTH study was observed within the time range in which the peak was recorded in the EMG‐A study in each subject. Such peak was never induced by electrical stimulation of the skin overlaying the ECR nerve in all the subjects.

The influence of TVS on the peak was examined in all 10 subjects. The peak diminished following TVS and was recovered 20–50 min (32 ± 13 min) after TVS cessation in all the subjects.

#### 3.2.3 Effects on the ADM motoneuron pool

The ECR nerve stimulation induced an early and significant peak (facilitation) in rectified and averaged EMGs of ADM in all 10 subjects (*p* < 0.05 for seven subjects; *p* < 0.01 for three subjects) (Table 4, Figure 7). The amount of facilitation was 11.6% ± 3.3%. The latency and duration of the peak were 24.6 ± 1.3 ms and 11.6 ± 3.3 ms, respectively. The peak in the PSTH study was observed within the period of the peak in the EMG‐A study in each subject. Such peak was never induced by electrical stimulation of the skin overlaying the ECR nerve in all the subjects.

The influence of TVS was examined in 6 out of 10 subjects. The peak diminished following TVS and was recovered 30–50 min (43 ± 7 min) after TVS cessation in all the subjects.

## 4 DISCUSSION

The present study evidenced the facilitation of motor units supplying APB, FDI, and ADM by low‐threshold afferents of ECR using the PSTH method. In addition, the ECR afferents stimulation facilitated rectified and averaged EMGs of these muscles as demonstrated using the EMG‐A method.

### 4.1 Effects from ECR to motoneurons innervating the intrinsic hand muscles

In the PSTH study, the conditioning stimulation of the ECR nerve with an intensity just below MT induced a peak in 14/64 (22%) APB, 15/41 (37%) FDI, and 14/38 (37%) ADM motor units of all the subjects. The remaining motor units were not affected. The conditioning stimulation also induced a peak in the EMG‐A study. Since the peak in the motor units was observed within the time window in which the peaks of the rectified and averaged EMGs were recorded, the peak in the EMG‐A study likely represented the facilitation of the motoneuron pool innervating each hand muscle (APB, FDI, and ADM). However, because the effects of conditioning stimulation were examined only during weak contraction in both the PSTH and EMG‐A studies, the facilitation was observed only for low‐threshold motor units. The effect on high‐threshold motor units is still unclear.

### 4.2 Afferent fibers responsible for the facilitation

For the afferent fibers responsible for the facilitation demonstrated in this study, group Ia afferents are most probably a strong candidate. The grounds are as follows. Firstly, we used electrical stimulation as conditioning stimulation. As the stimulus intensity was just below MT, low‐threshold afferents, that is, with thresholds lower than alpha motor fibers, were activated (Pierrot‐Deseilligny & Burke, 2012; Rothwell, 1994). In addition, the facilitation was not induced by stimulating the skin overlaying the ECR nerve in both PSTH and EMG‐A studies. These results suggest that the group Ia afferents, which originate from muscle spindles, and have the lowest threshold to electrical stimulation, are participating in the facilitation. However, the participation of group Ib afferents, which originate from Golgi tendon organs, and have similar diameters than that of Ia afferents, cannot be ruled out (Pierrot‐Deseilligny & Burke, 2012; Rothwell, 1994).

Secondly, we investigated the influence of TVS on the facilitation in the EMG‐A study. The facilitation was entirely depressed following TVS. Previous studies showed, using the microneurography technique, that many muscle spindles respond in a one‐to‐one manner to TVS of up to 100 Hz, and that some muscle spindles are driven by TVS of even higher frequencies. In contrast, secondary endings of muscle spindles and Golgi tendon organs are either insensitive or only slightly sensitive to TVS (Burke et al., 1976; Roll et al., 1989). In addition, we previously showed that TVS decreases the responsiveness of group Ia afferents from the muscle exposed to TVS (Nito et al., 2021). Indeed, a 100‐Hz TVS for 6 min decreased the monosynaptic facilitation mediated by group Ia afferents for more than 20 min after TVS removal. Animal experiments also supported the mechanism of depression by increasing the threshold of electrical activation of group Ia afferent (Coppin et al., 1970; Fetz et al., 1979; Hayward et al., 1986).

Considering these findings, group Ia afferents are likely responsible for the facilitation.

### 4.3 Central pathway

In the PSTH study, we estimated the central synaptic delay of the facilitation of motoneurons supplying intrinsic hand muscles in reference to the latency of the monosynaptic Ia facilitation induced by stimulating the homonymous nerve at the axilla. The difference between the central latency of the heteronymous facilitation and that of homonymous monosynaptic Ia facilitation was close to 0 ms. Because the central synaptic delay in humans takes about 1 ms (Petersen et al., 2002; Pierrot‐Deseilligny & Burke, 2012), our results were compatible with a monosynaptic path. Because the duration of the facilitation was sometimes ~6.2 ms, it may be claimed that not only monosynaptic but also non‐monosynaptic components could be responsible for the facilitation. However, duration of peaks in PSTH will be longer than the rise time of the excitatory post‐synaptic potentials (EPSP) when the induced EPSPs are not large (Fetz & Gustafsson, 1983). Therefore, at least the first component of facilitation was probably due to a monosynaptic path even if the duration of peaks in PSTH was longer.

### 4.4 Functional significance

The distribution pattern of group Ia afferents in muscles of the human upper limb has been extensively investigated (Aymard et al., 1995; Baldissera et al., 1983; Cavallari et al., 1992; Cavallari & Katz, 1989; Creange et al., 1992; Day et al., 1984; Katz et al., 1991; Kobayashi et al., 2016; Marchand‐Pauvert et al., 2000; Miyasaka et al., 2007; Naito et al., 1996, 1998; Nakano et al., 2014; Nito et al., 2016; Nito, Hashizume, Jimenji, et al., 2018; Nito, Hashizume, Suzuki, et al., 2018; Ogawa et al., 2005; Rossi et al., 1995; Sato et al., 2018; Shinozaki et al., 2017; Suzuki et al., 2005, 2012; Wargon et al., 2006). Based on these reports, it is known that the monosynaptic facilitation of the motoneurons innervating proximal muscles mediated by group Ia afferents from distal muscles (distal‐to‐proximal projections) are well developed in humans. Especially, monosynaptic facilitation from the ECR afferents to the motoneurons supplying the anterior part of deltoid (Creange et al., 1992), brachioradialis (Shinozaki et al., 2017), pronator teres has been investigated (Nakano et al., 2014), and the facilitation seems to be useful for stabilizing the proximal joints during wrist extension movement. However, no data regarding the proximal‐to‐distal projections have been reported. It is known that the proximal‐to‐distal projections are well developed in the baboon upper limb or the cat forelimb (Clough et al., 1968; Fritz et al., 1989). In cats, by stimulating the radial nerve trunk, the EPSP of group Ia afferents were induced in 4/18 (22%) motoneurons of intrinsic hand muscles innervated by the ulnar nerve. In contrast, the stimulation of the afferents from intrinsic hand muscles never induced EPSP in ECR motoneurons (Fritz et al., 1989). Similarly, in baboons, the stimulation of the radial nerve trunk induced EPSP in the motoneurons of intrinsic hand muscles innervated by the median (1/3, 33%) and ulnar nerves (1/12, 8%) (Clough et al., 1968). Because these studies examined the effects of stimulating the radial nerve trunk, which innervates the wrist and finger extensors, and because the sample size was very small, it is not appropriate to directly compare the percentages of facilitation. However, the present study shows that the ECR nerve stimulation induced monosynaptic Ia facilitation of APB, FDI, and ADM motoneurons in humans, indicating that the proximal‐to‐distal projections also exist in humans, who are capable of dexterous hand movements. In addition, the stimulation to the median and ulnar nerves at wrist induced monosynaptic Ia facilitation of ECR motoneurons in humans (Marchand‐Pauvert et al., 2000; Ogawa et al., 2005; Suzuki et al., 2005, 2012). Therefore, the distal‐to‐proximal projections might be human‐specific circuits. These differences might reflect the functional transition of neural networks from the forelimb to the upper limb in spinal cord.

Our previous work showed that a co‐contraction of ECR, thenar muscles, and hypothenar muscles was observed during hand‐grasping movement (Nito et al., 2016). Although the FDI is a prime mover during index finger abduction, FDI contraction was observed not only in the hand‐grasping movement but also in various grasping and pinch movements (Flament et al., 1993; Long II et al., 1970). In addition, the maximum grasping and pinch strength is obtained with the wrist in a slightly extended position (Halpern & Fernandez, 1996; Lamoreaux & Hoffer, 1995; Li, 2002; O'Driscoll et al., 1992; Pryce, 1980). Therefore, the facilitation demonstrated here might be involved in these hand movements. Similarly, we demonstrated that monosynaptic facilitation from the ECR afferents to FDS exists in humans, and the facilitation may also contribute to these movements (Nito et al., 2022). However, the facilitation to the motoneurons supplying intrinsic hand muscles occurred in 22%–37% of the cases in the PSTH study and amount of facilitation was 12%–15% (peak value) in the EMG‐A study, whereas the facilitation to FDS occurred in 60% of the cases in the PSTH study and amount of facilitation was 18% in the EMG‐A study (Nito et al., 2022), suggesting that the facilitation demonstrated here is weak compared to that to FDS. This weakness might allow dexterous hand movements. Descending commands from the brain affect strongly spinal motoneurons and change dynamically during hand movements (Lemon, 2008; Lemon et al., 1995). The facilitation described in this study might contribute, albeit moderately, to various hand movements by creating a situation where the excitability of motoneurons supplying intrinsic hand muscles can be increased easily.

## 5 CONCLUSIONS

The effects of low‐threshold afferents from ECR were investigated for individual motor units and motoneuron pools of APB, FDI, and ADM. The results indicate that a facilitation of the motoneurons supplying the intrinsic hand muscles is mediated by ECR group Ia afferents via a monosynaptic path in the human spinal cord.

## AUTHOR CONTRIBUTIONS

MN, MS, and AN conceived and designed research. MN, TY, WH performed experiments. MN, MS, AN analyzed data and interpreted results of experiments. MN, MS, AN drafted manuscript. All authors read and approved final version of manuscript.

## FUNDING INFORMATION

This research was supported by JSPS KAKENHI (Grant Number 19K19827, 22K17628) and Yamagata Health Support Society.

## CONFLICT OF INTEREST

The authors declare that they have no conflicts of interest.
